# β-Cyclodextrin as the Key Issue in Production of Acceptable Low-Cholesterol Dairy Products

**DOI:** 10.3390/molecules27092919

**Published:** 2022-05-03

**Authors:** Lukáš Kolarič, Petra Kántorová, Peter Šimko

**Affiliations:** Faculty of Chemical and Food Technology, Institute of Food Science and Nutrition, Slovak University of Technology in Bratislava, Radlinského 9, 812 37 Bratislava, Slovakia; xkantorovap@stuba.sk (P.K.); qsimko@stuba.sk (P.Š.)

**Keywords:** cholesterol, β-cyclodextrin, milk, dairy products, color, texture, functional foods, healthy foods

## Abstract

The application of cyclodextrins in food technology is extensive due to their unique ability to form complexes with many bioactive substances. Consumption of dairy products is associated with an increased risk of cardiovascular diseases (CVD) due to its high content of saturated fatty acids and cholesterol, so the production of low-cholesterol content products would be one of the critical steps in CVD prevention with regards to lowered total daily cholesterol intake. To maintain consumer acceptance, organoleptic profiles of such products should be, in the optimal case, the same with comparison to original ones. So, this study deals with the development of set low cholesterol foods (milk, cream, butter, soft cheese, cottage cheese) by β-cyclodextrin treatment and the characterization of their organoleptic profiles such as color and textural characteristics. During the experiments, high effectivity of cholesterol removal was reached as follows: milk–97.3%, cream–95.6%, butter–95.6%, cottage cheese–97.9%, soft cheese–97.7%, while color differences varied from 0.25 to 1.13 and textural characteristics were not influenced by cholesterol removal as well. So, it can be concluded that the proposed procedure is enabled to be substantial for the production of a new assortment of low-cholesterol dairy products with considerable health benefits toward the incidence of CVD.

## 1. Introduction

There is a significant improvement in primary prevention and medical treatment; however, cardiovascular diseases (CVD) are still the leading cause of mortality in the world, with around 1.8 million deaths in the European Union every year [1]. Since dietary habits negatively affect CVD, current diet guidance should focus on healthy dietary patterns such as the Mediterranean style and DASH (Dietary Approaches to Stop Hypertension), which emphasize foods that are relatively low in cholesterol content, such as fruits, vegetables, whole grains, low-fat or fat-free dairy products, etc. [2]. A recent observation by the European Society of Cardiology confirmed that the retention of low-density lipoprotein cholesterol (LDL-C) and other cholesterol-rich apolipoprotein B-containing lipoproteins within the arterial wall is the key initiating event in CVD [3]. As the intake of cholesterol in our body from food of animal origin is approximately 20–25% [4], another approach to reducing the risk of CVD can therefore be based on technological processes during raw material treatments, resulting in a reduced cholesterol content compared to the original matrix [5]. In fact, statistical analysis revealed that the application of cholesterol removal from milk and dairy products could decrease the total daily cholesterol intake in the diet from 369.8 mg to 296.3 mg, which is below the recommended value of 300 mg daily intake, set by health care officials in the Slovak Republic [5]. 

Milk and dairy products are an important source of nutrients, but in recent years dairy products have been associated with many negative health effects due to saturated fatty acid content, which can lead to increased levels of LDL-C and CVD risk [1]. According to Mazidi et al. [6], a population-based cohort study and meta-analysis revealed that there was no significant association between milk intake and total mortality, while a positive association was observed between milk intake and coronary heart diseases. Due to their high consumption, the importance of reducing the dietary cholesterol content in these products may play a role in the incidence of CVD occurrence. Several methods have been developed to reduce the cholesterol content in dairy, such as enzymatic conversion, steam distillation, supercritical extraction, or adsorption of various sorbents; however, these methods are mostly non-selective and negatively affect the nutritional and mechanical properties of the final products [7]. Cholesterol can also be removed by application of β-cyclodextrin (β-CD) since this procedure is sufficient to selectively remove cholesterol, while the content of other nutritional and flavor components is not significantly affected [8]. β-CD is a cyclic oligosaccharide consisting of seven glucose units with a doughnut-shaped molecule, which can entrap a cholesterol molecule almost perfectly due to its proper diameter [9]. The inclusion complex of cholesterol and β-CD is then comfortably separated by centrifugation [10]. Despite that, β-CD is inexpensive and has been classified as ‘safe’ by the Joint Expert Committee on Food Additives (JECFA) and the World Health Organization [9]. The efficiencies of cholesterol removal by β-CD in milk and dairy products are high, up to 98%; however, it depends on processing conditions such as mixing and centrifugation speed, the temperature of mixing, or settlement time as well as the concentration of β-CD [7,8]. 

According to the influence of cholesterol removal from milk and cream by β-CD on the nutritional and organoleptic aspects of low-cholesterol dairy products, several studies confirm the good selectivity and specificity of the process. Ha et al. [11] reported that the amounts of lactose trapped in β-CD did not exceed 0.03%, and other nutrients such as short-chain free fatty acids, free amino acids, and water-soluble vitamins also remained the same. According to Soni [12], the butter produced by cholesterol removal with β-CD is similar to the butter formed by the typical process in terms of flavor and consistency. From the point of rheological properties of the cheese, only slight differences were found between the low-cholesterol Camembert cheese and the control [8,13]. According to Elwahsh [14], the yield and main chemical components of cholesterol-reduced cheese and regular cream cheese were almost similar, while some changes in moisture, acidity, acid value, and pH were recorded during storage. Sensory evaluation of cholesterol-reduced milk obtained using β-CD revealed that 77% of consumers rated it as good or very good in terms of acceptance [10]. On the other hand, Nguyen et al. [15] stated that cholesterol removal by β-CD has a negative impact on the overall quality and consistency of butter, thus β-sitosteryl oleate should be used to improve these parameters. Furthermore, according to Galante et al. [16], the firmness values of the control soft cheese and the low cholesterol soft cheese were statistically different, because a weaker coagulum was formed as the cream base homogenization process caused a greater dispersion of fat globules in the curd and a reduction was achieved in the amount of free casein available from the casein network [8,16].

From recent observation, it is obvious that the elimination of cholesterol from milk and cream by the application of β-CD could play an important role in total cholesterol intake, but more studies are necessary to determine the influence on organoleptic and nutritional properties of low cholesterol dairy products. So, the purpose of this research was to prepare a set of low cholesterol dairy products and study their color and texture characteristics. 

## 2. Results and Discussion

### 2.1. The Production of Low-Cholesterol Dairy Products

From Table 1, it is evident that an excellent possibility of producing almost zero cholesterol milk or cream was confirmed. Treatment of milk with 1.0% (*w*/*w*) of β-cyclodextrin (β-CD) resulted in a reduction of 97.3% cholesterol content, while the highest reduction (98.1%) was achieved with 2.0% β-CD, but the results were not statistically different at *p* < 0.01. Similar results were noticed in the cream sample. The highest removal of cholesterol content (95.6%) was achieved with 5.0% of β-CD, but the results obtained by other β-CD concentrations were not statistically different at *p* < 0.01. 

The results are consistent with other studies, e.g., Zunnurain and Baig [17] reported that approximately 90% cholesterol was removed when milk was treated with 1.5% β-CD. According to the patent procedure [18], the treatment of raw milk or cream with 0.6% and 8.0% β-CD, respectively, resulted in a product where the final cholesterol content was not detected (the limit of detection was 2.6 mg/100 g). As the size of the β-CD cavity is ideal for the size of the cholesterol molecule, this procedure is highly selective for cholesterol; therefore, the treated products are very similar to conventional products [17,19]. 

The second part of the research was related to the production of low-cholesterol dairy products (butter, soft cheese, and cottage cheese) from the treated milk or cream sample. The results of the removal of cholesterol content are shown in Table 1. The reduction in cholesterol content is especially important in butter, as it consists of the highest amount of cholesterol (2483.44 mg/kg) compared to soft cheese (387.50 mg/kg) or cottage cheese (382.18 mg/kg). According to the OECD-FAO Agricultural Outlook 2019–2028 [20], around 30% of milk will be further processed into dairy products, while only butter production is projected to grow at 1.9% p.a., which is a faster rate than world milk production. Thus, for CVD prevention, the production of cholesterol-reduced butter could be a significant step. In fact, in this research, it is shown that by applying 5.0% β-CD, it was possible to produce butter with a cholesterol content of 108.66 mg/kg, which means a 95.6% reduction compared to the control sample. The results between the other concentrations of β-CD were not significantly different at *p* < 0.01. The process of elimination of cholesterol from the cream did not influence the yield of butter, as it was 34.0%, compared to 33.6% for standard cholesterol butter (Table 1). According to Alonso et al. [9], the process of cholesterol removal using β-CD is suitable for the manufacture of reduced-cholesterol butter with a final cholesterol content of 300.4 mg/kg (90% reduction compared to the control sample). According to Soni [12], a process for the preparation of reduced cholesterol content butter by applying beta-cyclodextrin to milk cream is highly efficient, with around 85 to 90% elimination of cholesterol from the butter. Dias et al. [21] did not notice significant differences between the concentrations of β-CD at 10, 15, and 20%, and suggested that 10% of β-CD is sufficient for the reduction of 90.7% in the cholesterol content in butter. They also investigated different complexation methods and found that the standard co-precipitation method is more effective than the kneading and physical mixture methods. 

Cheese production also increases every year. The highest percentage of total cheese consumption occurs in Europe and North America; however, cheese consumption is also supposed to grow where it was not traditionally part of the national diet, for example, in countries of Southeast Asia [20]. The cholesterol content in cheese is very variable depending on the microbiological and chemical composition of milk, cheese making technology, the ripening time, or the conditions of the cheese factory [22,23]. In a previous article [23], it was determined that the cholesterol content in different types of cheese varied from 548.18 to 1034.74 mg/kg. Thus, cheese consumption also contributes significantly to the total dietary intake of cholesterol. In this research, cheese samples were prepared by adding rennet (soft cheese) or by acid precipitation by citric acid (cottage cheese). The cholesterol content in the control cottage cheese (CCCh) was determined at 382.18 mg/kg, while the maximum reduction in cholesterol content (97.9%) was observed by the addition of 1.0% β-CD to milk (Table 1). The results were not significantly different at *p* < 0.01 among other β-CD concentrations. The CCCh yield was 33.9% and in low cholesterol cottage cheese (LCCCh) 32.5%. Control soft cheese (CSCh) contained 387.50 mg/kg cholesterol and the highest removal rate (97.7%) was achieved by applying 2.5% β-CD to milk, but also with 1.5% of β-CD, the reduction in cholesterol content was satisfying. The yield ranged from 25.1 to 28.6% (Table 1). It can be noticed that the final cholesterol content was lower in LCCCh samples than low cholesterol soft cheese (LCSCh). In general, soft cheese samples had higher dry matter content (around 35%) than cottage cheese samples (around 20%), thus the cholesterol content in LCSCh was relatively higher. In addition, the interaction between CD and host molecules is influenced by several factors, such as temperature, polarity, charge, or presented salts (calcium salts) which were added to milk in soft cheese production, could interrupt the CD-cholesterol complexes and thus released a small amount of cholesterol [24]. 

**Figure 1:**
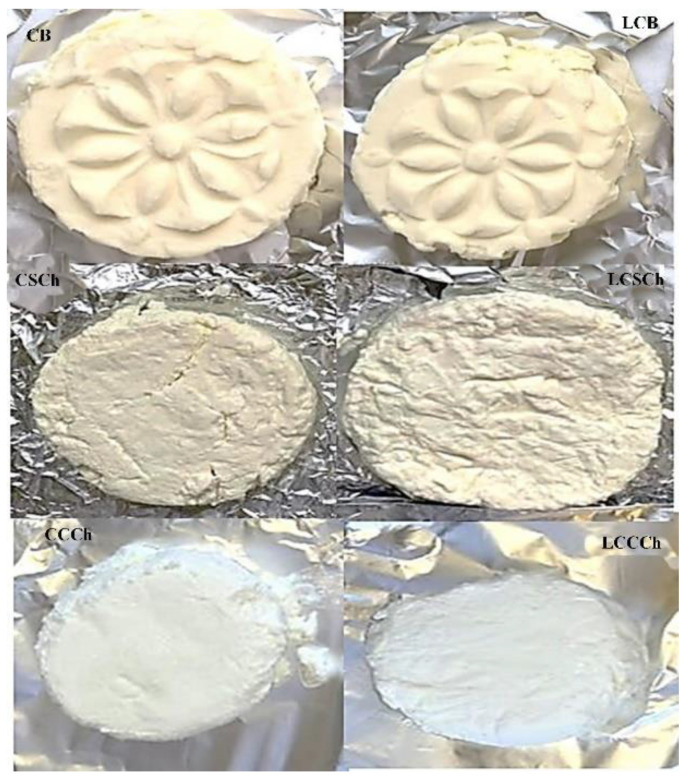
Pictures of produced dairy products. CB–control butter, LCB–low cholesterol butter, CSCh–control soft cheese, LCSCh–low cholesterol soft cheese, CCCh–control cottage cheese, LCCCh–low cholesterol cottage cheese.

Alonso et al. [25] produced experimental cheese with 1% of β-CD in milk and found that the cholesterol removal rate was 97.6%. According to them, treatment with β-CD is an effective process for the elimination of cholesterol from Manchego cheese, while preserving its nutritional properties. According to another study, the average reduction in cholesterol in soft cheese was 93% [16]. Han et al. [26] also determined similar values of the cholesterol removal rate in cream cheese, ranging from 91.4 to 91.8%, when milk was treated with 10% powdered or crosslinked β-CD. Through this process, it was also possible to produce low-cholesterol Camembert cheese with a chemical composition similar to that of the control and the cholesterol reduction in 90.6% [13], or Cheddar cheese, where the removal measure was lower (79.3%) with treatment with 1% β-CD [19]. Kwak et al. [27] reported that the homogenization pressure of milk significantly influenced the cholesterol removal rate in the Mozzarella cheese. The reduction in cholesterol content reached 68.43% with a pressure of 91 kg/cm^2^. Elwahsh [14] showed that regular cream cheese contained 946 mg/kg cholesterol, while treatment with 10% β-CD decreased it to only 84 mg/kg. The pictures of reduced-cholesterol dairy products are shown in Figure 1.

### 2.2. The Effect of β-CD Treatment on the Color Characteristics of the Final Products

**Figure 2:**
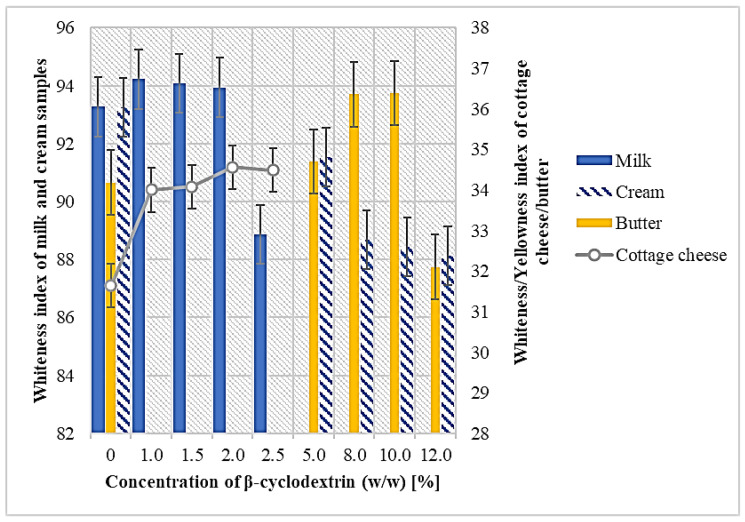
The effect of the concentration of β-cyclodextrin on the whiteness or yellowness index of milk products.

The color properties of food products are important, as color is related to the overall appearance and plays a significant role in food selection [28]. Milk is generally white in color as a consequence of light reflection by fat globules and other particles, such as calcium caseinate or colloidal calcium phosphate [29]. The results of the color coordinates for the milk and cream samples are shown in Table 2. The L* coordinate, which refers to the lightness values of the control milk and cream sample was determined at 97.46 and 97.61, respectively. Milovanovic et al. [28] mentioned that L* ranged from 82.2 (cow milk) to 84.9 (pasteurized and sterilized milk), and Gianni et al. [10] determined L* for raw milk at 91.61. According to an ANOVA analysis, significant differences at *p* < 0.01 were found between control milk and reduced cholesterol milk in all coordinates. However, the total color differences (Δ*E*) were less than five units, indicating that no significant differences are perceptible to consumers by visual observation [30]. In the cream samples, a significant difference was found at *p* < 0.01 in a* coordinate but Δ*E* was still irrelevant (0.25). The whiteness index (WI) increased from 93.27 (0% β-CD) to 94.22 (1.0% β-CD) and in the cream sample dropped from 93.25 (0% β-CD) to 88.12 (12.0% β-CD). The results of the effect of cholesterol removal by different concentrations of β-CD from milk and cream on WI are shown in Figure 2. According to Gianni et al. [10], the addition of 0.6% β-CD had no effect on L*, a*, and b* values in raw or homogenized milk. From the results of this research, it could be suggested that lower concentrations of β-CD are preferred according to the final color. In fact, these concentrations are still sufficient for cholesterol removal above 95% (Table 1), therefore higher amounts of β-CD are not required. 

The results of the effect of cholesterol removal on the color coordinates for the butter and cheese samples are shown in Table 2. As in the case of milk or cream, β-CD concentration also influenced the color coordinates in dairy products, however, at the lowest investigated concentrations, the differences were insignificant. L* values of butter increased from 72.45 (0% β-CD) to 73.03 (5% β-CD), but the yellowness index remained the same. Δ*E* value of 5% β-CD low cholesterol butter (LCB) was only 0.57, but in the 12% β-CD LCB it was 17.07, thus visual differences were observed compared to the control butter (CB). Bhatia et al. [31] reported a lower L* value for low-cholesterol ghee (47.95 compared to 50.08 for control ghee), but according to them, the cholesterol removal process did not affect the typical color of cow ghee to the extent that it could affect consumer acceptance. According to Kim et al. [32], there was a significant difference in L* values between CB and LCB. LCB samples were whiter than the control; however, they used crosslinked β-CD. L* value for CCCh was 83.25 and 1% β-CD LCCCh indicated 82.94, thus it was not significantly affected by cholesterol removal. Δ*E* between CCCh and 1% β-CD LCCCh was only 0.27, so visually they were similar. The same trend was also observed in CSCh. L* values in the soft cheese samples decreased from 80.38 (0% β-CD) to 79.32 (1.5% β-CD), but Δ*E* was only 1.13. The whiteness index of the cottage cheese samples remained the same. The results of β-CD treatment on the whiteness and yellowness index of cottage cheese and butter, respectively, are shown in Figure 2. From the results of the color analysis of dairy products, it can be concluded that the amount of used β-CD influenced the final color properties, but was more pronounced in butter than cheese. As butter consists of a higher fat content, the variation in the size or geometry of fat globules after cholesterol removal may be an explanation for this phenomenon [28]. Therefore, it could be recommended to apply lower concentrations of β-CD, which are still sufficient for a cholesterol removal rate greater than 95%. 

The correlation analysis confirmed that a strong positive correlation was found between L* values and the cholesterol content in milk (*r* = 0.95), L* values and the cholesterol content in cream (*r* = 0.80), a* values and the cholesterol content in cottage cheese (*r* = 0.88) and L* values and the cholesterol content in soft cheese (*r* = 0.80). A strong negative correlation was observed only between b* values and the cholesterol content in milk (*r* = −0.86) and Δ*E* and the cholesterol content in milk (*r* = −0.85).

### 2.3. The Effect of β-CD Treatment on the Texture Characteristics of the Final Products

**Figure 3:**
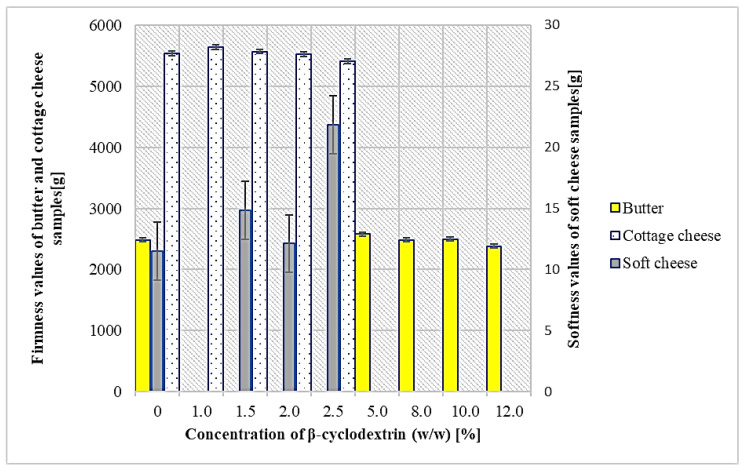
The effect of the concentration of β-cyclodextrin on the firmness and softness values of cottage cheese, butter, and soft cheese, respectively.

The results of the effect of cholesterol removal on the texture of the final products are shown in Table 3 and Figure 3. The firmness value of the control milk sample was 33.73 g and after cholesterol removal, it increased to 34.17. However, the differences between the control and treated samples were not significant at *p* < 0.01, thus the β-CD treatment had no effect on milk firmness. The same results were noticed according to the consistency of milk. The firmness value of the control cream sample was determined at 33.79 g and decreased to 33.22 g, but the differences were also insignificant. No published article related to the determination of textural changes after cholesterol removal from milk or cream was found, but according to Shim et al. [33], the β-CD treatment process for cholesterol removal did not show a profound adverse effect on the functional properties of cream after whipping, such as apparent viscosity, overrun, foam instability, or de-emulsification. 

The results of the measurement of the firmness values of the butter and cottage cheese samples and the softness values of the soft cheese samples are shown in Figure 3. There were no significant differences at *p* < 0.01 between the CB and LCB samples. The firmness of the CB was determined at 2482.24 g and only increased to 2583.06 g. According to Nguyen et al. [15], the CB samples were significantly firmer and more adhesive than the LCB samples. They used 15% of β-CD in the removal of cholesterol from the cream, so perhaps this caused a reduction in the firmness of the LCB. According to this research, 5% of β-CD is effective enough for the successful elimination of cholesterol from cream above 95%, so in practical use, it is not necessary to work with higher β-CD concentrations. The study published by Kim et al. [32] showed that treatment with β-CD increased the hardness, elasticity, and cohesiveness of butter, so they suggested the addition of evening primrose oil and phytosterols, which resulted in lower values for these parameters.

The firmness of CCCh and LCCCh was also not significantly influenced by β-CD treatment. It increased only from 5542.44 (0% β-CD) to 5640.07 with insignificant differences at *p* < 0.01. The softness between the CSCh and LCSCh samples was similar, except for the application of 2.5% β-CD, which indicated the highest softness value (21.85 g), but the dry matter of this sample was also the highest (37.2% compared to 32.5% for CSCh), so it could cause this difference. According to Galante et al. [16], the firmness of soft cheese without treatment was statistically higher compared to cholesterol-reduced cheese, which could be explained by weak coagulum formation. However, Kim et al. [13] found no significant differences between the control and cholesterol-reduced cheese samples in all rheological characteristics, such as hardness, cohesiveness, springiness, gumminess, or brittleness. According to Kwak et al. [27], mozzarella cheese made with homogenized milk and treated with 1% β-CD exhibited significantly lower hardness, gumminess, and chewiness, but higher cohesiveness and elasticity values compared to the control cheese. Jung et al. [34] found that cholesterol-reduced Gouda cheese had no adverse effects compared to the control on texture properties during 6 months of ripening. As cheese products are a very large and versatile group, the results in textural properties are also very variable. 

The correlation analysis did not reveal any strong positive or negative correlation between the textural properties and the cholesterol content in dairy products.

## 3. Materials and Methods

### 3.1. Materials and Reagents

Cream (40% fat content), homogenized pasteurized cow milk (3.5% fat content), and citric acid were purchased from a local market. β-cyclodextrin (β-CD) (fine chemical) was obtained from Cyclolab (Cyclodextrin Research and Development Laboratory Ltd., Budapest, Hungary). Liquid rennet (100% chymosin) and calcium chloride (saturated solution 33%) were purchased from Milchema s.r.o. (Považská Bystrica, Slovakia). Potassium hydroxide (KOH), chloroform, n-hexane, ethanol, and anhydrous sodium sulfate were of analytical grade and were obtained from Centralchem s.r.o. (Bratislava, Slovakia). Methanol and acetonitrile for HPLC analysis (Fisher Chemical, Loughborough, UK) were of HPLC grade.

### 3.2. The Preparation of Low Cholesterol Milk and Cream Base

Cholesterol removal by β-CD was performed according to Kolarič and Šimko [35]. For the preparation of the milk used for the production of cheese, the following conditions were applied: 1 L of pasteurized milk (3.5% fat content) was mixed with 4 different levels of β-CD (1.0, 1.5, 2.0, and 2.5% *w*/*w*) at 840 rpm, 25 °C and for 20 min. The milk was then settled for 120 min at 4 °C and finally centrifuged at 70× *g* for 20 min due to the separation of the β-CD-cholesterol complex. For the preparation of the cream base used for the production of butter, the following conditions were applied: 200 mL of cream (40% fat content) was mixed with β-CD (5.0, 8.0, 10.0, and 12.0% *w*/*w*) at 480 rpm, 40 °C, and for 20 min. The treated cream was settled for 30 min at 4 °C and centrifuged at 450× *g* for 20 min. The mixtures were stirred with a magnetic stirrer (Arex-6 Connect Pro, Velp Scientifica, Usmate, Italy) and centrifuged in a laboratory centrifuge (Hettich Zentrifugen, Tuttlingen, Germany).

### 3.3. Production of Low Cholesterol Dairy Products

The manufacture of butter was carried out according to Nguyen et al. [15]. Five different types of butter were produced: a control butter (CB) was made from standard cream (40% fat content) and four low cholesterol butter samples (LCB) were made from cream treated with β-CD at 5.0, 8.0, 10.0, and 12.0% (*w*/*w*), as described in Section 2.2. Each cream was churched with an electric blender (Hyundai Hand Mixer, Soul, Korea) until butter granules became visible. The buttermilk was separated on the sieve, and the butter was washed twice with cold tap water. Finally, the butter was molded, weighed, and placed in containers for further analysis. 

Cottage cheese was made by heat acid precipitation with citric acid according to the modified procedure of Chakraborty et al. [36]. A control cottage cheese (CCCh) was made from standard pasteurized cow milk (3.5% fat content) and four low cholesterol cottage cheese samples (LCCCh) were made from milk treated with 1.0, 1.5, 2.0, and 2.5% (*w*/*w*) of β-CD, as described in Section 2.2. To 1 L of milk, approximately 350 mL of citric acid solution (2% *w*/*v*) was added until the pH value reached 4.5. The milk was settled for 3 h in the laboratory temperate and the coagulation was completed by heating to 60 °C. The coagulated mass was left undisturbed with whey for 10 min. The coagulum was separated using a gauze cloth to the cheese molds and the cottage cheese was left there until the next day for complete drainage of the whey. Finally, cottage cheese was collected and stored in the refrigerator for further analysis. 

Soft cow cheese was prepared according to the modified procedure by Elsamani et al. [37]. Low cholesterol soft cheese samples (LCSCh) were made from pasteurized milk treated with 1.5, 2.0, and 2.5% (*w*/*w*) of β-CD, and control soft cheese (CSCh) from standard cow milk (3.5% fat content). The milk was heated to 40 ± 5 °C and then 1.0 mL of saturated CaCl_2_ solution (33%) and 0.2 mL of liquid rennet (100% chymosin) were added. The mixture was left for coagulation for 1 h. The coagulated curd was cut to separate the whey into pieces in a dimension of 2 × 2 cm and after 10 min to smaller pieces. After another 10 min, the cheese curd was poured into a mold lined with a gauze cloth and left to settle in the refrigerator overnight. The cheese was removed from the mold and stored in the refrigerator for further analysis.

The yield of the butter, cottage cheese samples, and soft cheese was expressed as [36]:

(1) $$\%Yield=\frac{Mass\;of\;produced\;product}{Mass\;of\;milk\;or\;cream}\times 100$$

### 3.4. Analysis of Cholesterol Content

The analysis of cholesterol content was carried out according to Kolarič and Šimko [35]. The samples (5.0, 1.0, and 0.5 g for milk, cream or cheese, and butter, respectively) were refluxed with 15 mL of 1 mol·L^−1^ KOH methanolic solution for 15 min. Then, cholesterol extraction was performed with the mixture of *n*-hexane and chloroform (1:1, *v*/*v*) in duplicate. The extracts were filtered through anhydrous Na_2_SO_4_, evaporated using a rotary vacuum evaporator (Witeg, Wertheim, Germany), and the residue was dissolved in 3 mL of methanol, filtered using a PTFE filter with a membrane of 0.2 μm membrane (Agilent Technologies, Santa, Clara, CA, USA), and analyzed by HPLC.

The HPLC system (Agilent Technologies 1260 infinity system, Santa Clara, CA, USA) was equipped with a vacuum degasser, a quarterly pump, an autosampler, and the UV–DAD detector was set at 205 nm. The isocratic elution was carried out at a flow rate of 0.5 mL.min^−1^ using the mobile phase consisting of acetonitrile and methanol 60:40 (*v*/*v*). The injection volume was 10 µL. The stationary phase consisted of the Zorbax Eclipse Plus C_18_ column (2.1 × 50 mm, 5 μm particle size) and the Zorbax SB-C_18_ guard column (4.6 × 12.5 mm, 5 μm particle size). The retention time of cholesterol was 2.2 min. The results were recorded using OpenLab CDS software, ChemStation Edition for LC, and LC/MS systems (product version A.01.08.108) [35].

### 3.5. Texture Evaluation

The texture evaluation of the milk and cream base as well as final dairy products was characterized by the TA.XT plus texture analyzer (Godalming, Surrey, UK) using a Peltier cabinet. For the determination of the firmness [g] and consistency [g.s.] of treated milk and cream samples, the penetration test was used in a compression mode to measure force using a 36 mm cylinder probe (P/36R) with a 5 kg load cell, test speed at 1.00 mm/s, and target distance at 10.0 mm. The firmness values [g] of CB, LCB, CCCh, and LCCCh samples were evaluated using the spreadability test in a mode of measured force in compression using the TTC Spreadability Rig (HDP/SR), the test speed at 3.0 mm/s, and the target distance at 23 mm for the CB and LCB samples or 25 mm for the CCCh and LCCCh samples. The measurement was carried out in the Peltier cabinet at constant temperatures of 5 °C and 10 °C for the butter and cottage cheese samples, respectively, which were controlled by the Peltier control unit. The softness [g] values of the CSCh and LCSCh samples were measured using the penetration test for the softness of cheese in a compression mode measure force using a 3 mm cylinder probe (P/3) with a 5 kg load cell, test speed at 1.0 mm/s, and target distance at 10.0 mm. The results were recorded using Exponent Stable MicroSystem software (version 6.1.4.0). 

### 3.6. Characterization of Color Properties

The color of the milk and cream base as well as the final products were measured using a Cary 300 UV-VIS spectrophotometer (Agilent Technologies, Santa Clara, CA, USA) with a DRA-CA-30I (internal) sphere accessory. The color was evaluated using the CIEL*a*b* coordinate system with respect to the illuminant D65 and a visual angle of 10°. The measured parameters were L* (lightness factor), a* (−a* = green, +a* = red), b* (−b* = blue, +b* = yellow), WI (whiteness index), and YI (yellowness index). The spectrophotometer was standardized with a white plate supplied with the equipment. Δ*E* was calculated to estimate the visual differences between control samples and low cholesterol samples according to Equation (2) [6]:

(2) $$\Delta E=\sqrt{{\left(\Delta L^{*}\right)}^{2}+{\left(\Delta a^{*}\right)}^{2}+{\left(\Delta b^{*}\right)}^{2}\;}\;\;$$

The results were recorded with the Cary WinUV software (version 4.20 (468)).

### 3.7. Statistical Evaluation

Results are expressed as mean ± standard deviation or as a percentage. Statistical analysis was performed using Microsoft Excel 365 (version 2012). Data were subjected to a Kolmogorov–Smirnov test to check normality and one-way analysis of variance (ANOVA) with Tukey’s comparison test. Values were considered significantly different when *p* < 0.01. The results were at least triplicated. A correlation analysis was performed to determine the type of dependence between all measured parameters of the low cholesterol content samples and the control samples. The level of association was quantified using Pearson’s correlation coefficients (*r*). A high correlation was confirmed when *r* was between 0.80 and 1.0 [38].

## 4. Conclusions

The association of high dietary cholesterol intake with the incidence of CVD is well documented in many studies. In this study, a promising approach was shown to reduce CVD risk by lowering the cholesterol content in various dairy products. Treatment of cow milk and cream with β-CD was effective in cholesterol removal of 97.3% and 95.6%, respectively. Then, these materials with almost zero cholesterol content could be used to produce dairy products with the same yield as the products prepared by standard procedure. The cholesterol content in butter was reduced to 95.6%, in cottage cheese to 97.9%, and in soft cheese to 97.7%. In general, cholesterol-reduced products maintained their color and textural properties. Total color differences (Δ*E*) were varied only between 0.25 and 1.13, but it was affected by the concentration of β-CD. The textural properties of reduced cholesterol milk, cream, and dairy products were not statistically different *p* < 0.01 from the control samples, as well. Thus, the cholesterol removal did not influence these important characteristics of the products due to overall acceptability. The production of such products can play an important role in the modern healthy diet in reducing CVD risk.

## Figures and Tables

**Table 1:** The effect of the concentration of β-CD (*w*/*w*) on the removal of cholesterol content in dairy products.

	β-CD Concentration (%)	Cholesterol Content (mg/kg) ^1^	Cholesterol Removal (%) ^1^	Yield (%)
Milk (pasteurized, 3.5% fat content)	0.0	134.54 ± 0.75	-	-
	1.0	3.68 ± 0.45	97.3 ± 0.3	-
	1.5	3.14 ± 0.12	97.7 ± 0.1	-
	2.0	2.57 ± 0.29	98.1 ± 0.2	-
	2.5	3.33 ± 0.11	97.5 ± 0.1	-
Cream (pasteurized, 40% fat content)	0.0	1227.64 ± 15.60	-	-
	5.0	108.31 ± 6.64	95.6 ± 0.7	-
	8.0	149.01 ± 14.18	94.0 ± 0.6	-
	10.0	165.79 ± 11.64	93.3 ± 0.5	-
	12.0	159.03 ± 13.91	93.6 ± 0.9	-
Butter	0.0	2483.44 ± 30.34	-	33.6
	5.0	108.66 ± 10.37	95.6 ± 0.4	34.0
	8.0	136.99 ± 8.55	94.5 ± 0.3	31.0
	10.0	118.44 ± 13.51	95.2 ± 0.5	33.0
	12.0	72.77 ± 1.44	96.7 ± 0.5	33.5
Cottage cheese	0.0	382.18 ± 10.40	-	33.9
	1.0	8.09 ± 1.96	97.9 ± 0.5	32.5
	1.5	13.38 ± 0.84	96.5 ± 0.2	29.5
	2.0	12.56 ± 1.59	96.7 ± 0.4	30.3
	2.5	10.97 ± 1.33	97.7 ± 0.4	33.7
Soft cheese	0.0	387.50 ± 18.06	-	28.6
	1.5	71.28 ± 0.57 *	81.6 ± 0.2 *	27.1
	2.0	47.29 ± 2.68 *	87.8 ± 0.7 *	25.5
	2.5	9.09 ± 0.88	97.7 ± 0.2	25.1

^1^ Results are expressed as mean ± standard deviation and *n* = 3. * The results are statistically different at *p* < 0.01; CD, cyclodextrin.

**Table 2:** The effect of the concentration of β-CD on the color characteristics of dairy products. Color coordinates are expressed as L* (lightness factor), a* (−a* = green, +a* = red), b* (−b* = blue, +b* = yellow) and Δ*E* (color differences).

	Concentration of β-CD (%)	L* ^ 1^	a* ^ 1^	b* ^ 1^	Δ*E*
Milk (pasteurized, 3.5% fat content)	0.0	97.46 ± 0.04	0.12 ± 0.02	0.36 ± 0.03	-
	1.0	^+^ 96.87 ± 0.03	^+^ 0.06 ± 0.01	^+^ 0.48 ± 0.01	0.37
	1.5	^+^ 96.79 ± 0.03	^+^ 0.05 ± 0.01	^+^ 0.50 ± 0.01	0.47
	2.0	^+^ 96.77 ± 0.03	^+^ 0.05 ± 0.01	^+^ 0.47 ± 0.02	0.49
	2.5	^+^ 96.61 ± 0.04	0.16 ± 0.01	^+^ 0.57 ± 0.01	0.77
Cream (pasteurized, 40% fat content)	0.0	97.61 ± 0.06	0.12 ± 0.01	0.27 ± 0.02	-
	5.0	97.17 ± 0.02	^+^ 0.17 ± 0.00	0.28 ± 0.01	0.25
	8.0	^+^ 96.44 ± 0.11	^+^ 0.03 ± 0.00	^+^ 0.56 ± 0.05	1.55
	10.0	^+^ 96.63 ± 0.03	0.11 ± 0.01	^+^ 0.68 ± 0.04	1.30
	12.0	^+^ 96.71 ± 0.02	^+^ 0.15 ± 0.00	^+^ 0.80 ± 0.02	1.31
Butter	0.0	72.45 ± 0.88	0.58 ± 0.04	15.25 ± 0.23	-
	5.0	73.03 ± 0.39	0.69 ± 0.06	15.70 ± 0.31	0.57
	8.0	^+^ 74.80 ± 0,80	0.59 ± 0.06	^+^ 16.81 ± 0.31	7.96
	10.0	^+^ 74.54 ± 0.47	^+^ 0.97 ± 0.05	^+^ 16.75 ± 0.14	6.77
	12.0	^+^ 76.37 ± 0.45	^+^ 0.95 ± 0.08	^+^ 16.50 ± 0.07	17.07
Cottage cheese	0.0	83.25 ± 0.59	0.38 ± 0.02	6.07 ± 0.04	-
	1.0	82.94 ± 0.43	^+^ 0.22 ± 0.02	^+^ 5.98 ± 0.09	0.27
	1.5	^+^ 85.77 ± 0.15	^+^ 0.26 ± 0.02	6.16 ± 0.15	6.37
	2.0	84.67 ± 0.54	^+^ 0.27 ± 0.02	5.72 ± 0.25	2.15
	2.5	83.99 ± 0.72	^+^ 0.30 ± 0.01	5.71 ± 0.23	0.68
Soft cheese	0.0	80.38 ± 0.34	1.91 ± 0.04	8.67 ± 0.07	-
	1.5	79.32 ± 0.33	1.82 ± 0.02	8.65 ± 0.17	1.13
	2.0	^+^ 78.52 ± 0.41	^+^ 1.38 ± 0.05	^+^ 6.70 ± 0.18	7.63
	2.5	^+^ 76.64 ± 0.11	^+^ 1.56 ± 0.03	^+^ 7.26 ± 0.12	2.67

^1^ The results are expressed as mean ± standard deviation and *n* = 4. ^+^ The results are statistically different at *p* < 0.01 from the control sample (0.0% β-CD); CD, cyclodextrin.

**Table 3:** The effect of the concentration of β-CD on the textural characteristics of milk and cream.

	Concentration of β-CD (%)	Firmness (g) ^1^	Consistency (g.s.) ^1^
Milk (pasteurized, 3.5% fat content)	0.0	33.73 ± 0.18	245.10 ± 1.20
	1.0	34.17 ± 0.14	245.82 ± 1.37
	1.5	34.37 ± 0.09	246.16 ± 2.04
	2.0	34.12 ± 0.22	244.63 ± 1.32
	2.5	34.06 ± 0.27	245.06 ± 0.88
Cream (pasteurized, 40% fat content)	0.0	33.79 ± 0.13	243.92 ± 0.57
	5.0	33.22 ± 0.07	240.82 ± 0.68 *
	8.0	33.21 ± 0.30	239.95 ± 1.36 *
	10.0	33.24 ± 0.04	240.82 ± 0.68 *
	12.0	33.00 ± 0.17	238.93 ± 0.90 *

^1^ The results are expressed as mean ± standard deviation and *n* = 4. * The results are statistically different at *p* < 0.01; CD, cyclodextrin.

## Data Availability

The data that support the findings of the study are available from the corresponding author upon request.

## Sample Availability

Samples of the materials and products are available from the authors.
